# TRIM31: A molecule with a dual role in cancer

**DOI:** 10.3389/fonc.2022.1047177

**Published:** 2022-12-22

**Authors:** Yafei Guo, Ping Lin, Yimin Hua, Chuan Wang

**Affiliations:** ^1^ Key Laboratory of Birth Defects and Related Diseases of Women and Children of MOE, Department of Pediatrics, West China Second University Hospital, Sichuan University, Chengdu, China; ^2^ The Cardiac Development and Early Intervention Unit, West China Institute of Women and Children’s Health, West China Second University Hospital, Sichuan University, Chengdu, Sichuan, China; ^3^ Lab of Experimental Oncology, State Key Laboratory of Biotherapy and Cancer Center, West China Hospital, Sichuan University, Chengdu, China

**Keywords:** TRIM31, cancer, oncogene, tumor suppressor, innate immunity

## Abstract

Tripartite motif (TRIM) 31 is a new member of the TRIM family and functions as an E3 ubiquitin ligase. Abnormal TRIM31 expression leads to a variety of pathological conditions, such as cancer, innate immunity diseases, sepsis-induced myocardial dysfunction, cerebral ischemic injury, nonalcoholic fatty liver disease and hypertensive nephropathy. In this review, we comprehensively overview the structure, expression and regulation of TRIM31 in cancer. Moreover, we discuss the dual role of TRIM31 in human cancer, and this dual role may be linked to its involvement in the selective regulation of several pivotal cellular signaling pathways: the p53 tumor suppressor, mTORC1, PI3K-AKT, NF-κB and Wnt/β-catenin pathways. In addition, we also discuss the emerging role of TRIM31 in innate immunity, autophagy and its growing sphere of influence across multiple human pathologies. Finally, a better understanding of the dual role of TRIM31 in cancer may provide new therapeutic strategies aimed at inhibiting the cancer-promoting effects of TRIM31 without affecting its tumor suppressor effects.

## 1 Introduction

Ubiquitination is a common posttranslational modification of proteins. It participates in various cellular processes and physiological responses in cancer, inflammatory disorders, infection and other diseases by regulating the degradation and activation of intracellular proteins (1). The ubiquitination process is catalyzed by E1, E2 and E3, among which E3 ubiquitin ligase is mainly involved in the recognition and binding of target proteins (2). E3 ubiquitin ligases can be divided into two major classes: homologous to E6-AP COOH terminal (HECT) E3 ubiquitin ligases and RING finger-containing E3 ubiquitin ligases. Although TRIMs are considered to be RING finger-containing E3 ubiquitin ligases, not all TRIM E3 ubiquitin ligases have a RING domain. To date, there are 9 ring-domain-free TRIM proteins in humans (3). Apart from the RING finger domain, TRIM proteins also contain one or two zinc-binding motifs, named B-boxes, and a coiled-coil domain. According to their domains, TRIM proteins are divided into I to XI subfamilies (4–6). TRIM proteins regulate important cellular processes, such as intracellular signal transduction, innate immunity, transcriptional regulation, autophagy, and carcinogenesis (7, 8). In cancer research, TRIM members act as oncogenes or tumor suppressor genes in ovarian cancer, renal cell carcinoma, gastric cancer, and breast cancer by controlling multiple processes such as transcriptional regulation, DNA repair, cell proliferation, apoptosis, and metastasis (9–13).

**Figure 1:**
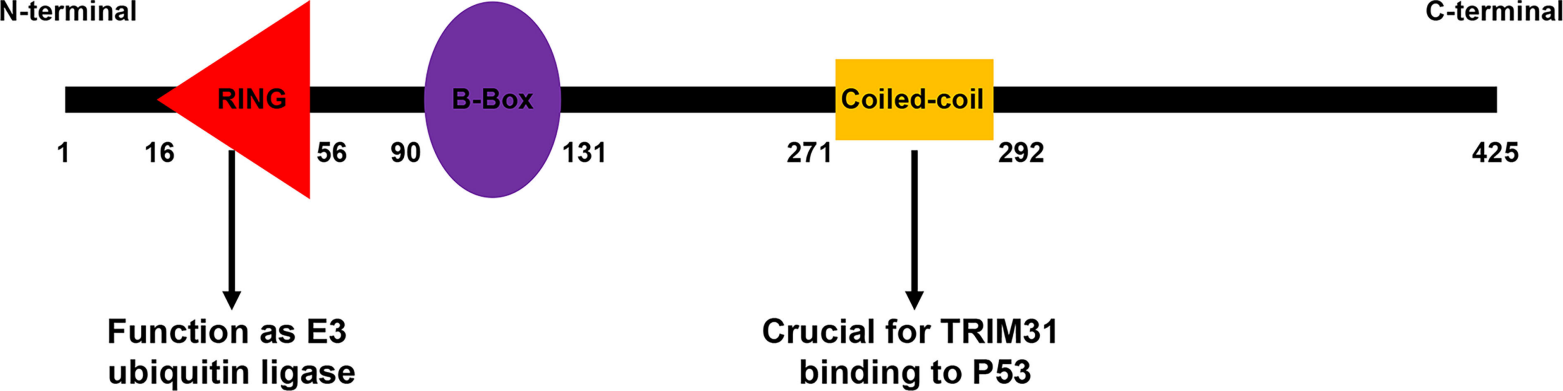
Domain organization of the TRIM31 protein. The different TRIM31 domains are reported with numbers and indicate the first and last amino acids of each domain.

TRIM31 is a member of the TRIM family, and structural analysis found that it contains a RING domain, which makes it an E3 ubiquitin-protein ligase (14). The RING domain is a zinc-binding motif located in amino acids 10-20 of the first methionine in nearly all TRIM protein N-terminal portions (15). General insights into RING domain function are derived from the report that the RING domain contains the CBL protein, which has shown that the RING domain regulates ubiquitination events (16–18). It has been reported that C16, C36, C53, C56, and C58 are the key amino acids of the RING domain, and mutation of these amino acid sites can inhibit the E3 ubiquitin ligase activity of TRIM31 (19–21). Apart from the RING domain, TRIM31 also contains one zinc-finger domain named the B box (type 2 box). B-box domains exist in more than 1500 proteins from a variety of organisms, and they can be divided into two groups, in which the intervals of 7-8 zinc binding residues of type 1 and type 2 B-box domains are different. Type 2 B-box proteins play a role in the ubiquitination process. After the B boxes are the coiled-coil region at the N-terminus, this domain regulates homomeric and heteromeric interactions between TRIM proteins and other proteins, especially self-association. In our research, the coiled-coil region was important for the binding of TRIM31 and p53 (22). TRIM31 has no domain at the C-terminus (**Figure 1**).

Recent studies on TRIM31 strongly advocate for the critical role of TRIM31 in cancer, immunity and inflammation. In this review, in addition to accumulating recent corroborations that endorse this dual role of TRIM31 in cancer, we also discuss the emerging role of TRIM31 in innate immunity, inflammation and autophagy and its growing sphere of influence across multiple other human pathologies.

## 2 Expression of TRIM31 in cancer and its clinical value

**Figure 2:**
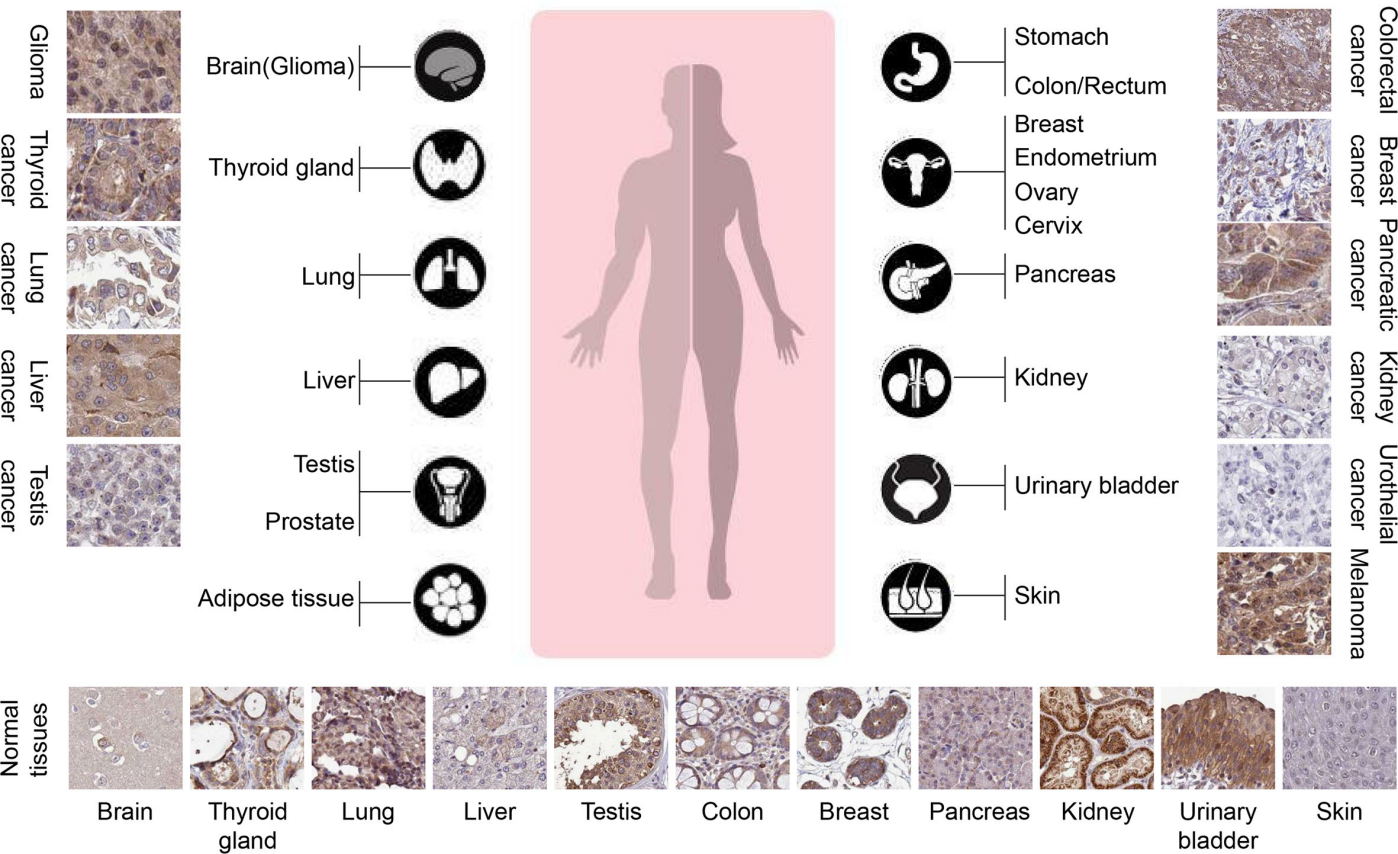
TRIM31 is highly expressed in several types of tumors (The Human Protein Atlas data). The rabbit polyclonal antibody HPA046400 (Sigma Aldrich) was used for the immunohistochemistry assay. The cancer tissues of glioma, melanoma, thyroid gland, pancreatic cancer and liver cancer were strongly cytoplasmic stained. Occasional membranous positivity was observed in colorectal cancer. Urothelial cancer and most kidney cancers were negatively stained. Normal tissues showed weak to strong cytoplasmic positivity with HPA046400 antibody, as presented at the bottom. In glioma, thyroid cancer, liver cancer, colorectal cancer, and pancreatic cancer, the expression of TRIM31 in cancer cell cytoplasm is higher than that in adjacent normal tissues. However, in lung cancer, testis cancer, kidney cancer, and urothelial cancer, the expression of TRIM31 was lower than that in adjacent normal tissues.

To date, the clinical correlation of TRIM31 in cancer is still elusive. Several reports have revealed that there is a positive correlation between the expression of TRIM31 and cancer prognosis in specific cancer types. TRIM31 is upregulated in gastric adenocarcinoma and may be a potential biomarker of gastric cancer because it is overexpressed in the precancerous stage (23). TRIM31 was markedly upregulated in hepatocellular carcinoma, gallbladder cancer, colorectal cancer, high-grade glioma, pancreatic cancer and acute myeloid leukemia, and the high expression of TRIM31 was also associated with an aggressive phenotype, advanced disease status and poor prognosis (24–29). Multivariate survival analysis demonstrated that TRIM31 was an independent prognostic factor for glioma patients (27). From the Human Protein Atlas data, immunohistochemical analysis found that TRIM31 was more highly expressed in liver, gastric, pancreatic, gallbladder, colorectal tumors and glioma (**Figure 2**, more information please see www.proteinatlas.org). It is suggested that upregulation of TRIM31 is a common feature of many epithelial cancers and predicts a poor prognosis. However, several reports have indicated that TRIM31 is downregulated in cancer; for example, TRIM31 expression is downregulated in lung cancer tissues and cell lines and correlates with clinic-pathological factors (30). Our research showed that TRIM31 expression was decreased in breast cancer tissues and that lower TRIM31 levels were associated with worse survival of breast cancer patients (22). Altogether, the aforementioned studies showed that TRIM31 was upregulated in liver, gastric, pancreatic, gallbladder, colorectal tumors and glioma, and higher levels of TRIM31 are related to the poor prognosis of cancer patients. However, there are also opposite conclusions. TRIM31 was downregulated in lung and breast cancer, and higher expression of the TRIM31 gene is linked to better overall survival of patients.

## 3 Regulation of TRIM31 expression

The expression of TRIM31 in diverse cancer cells is different, and its expression is tightly controlled by various factors. Recent reports have shown that TRIM31 expression is regulated by retinoid, microRNA and posttranslational modifications. Retinoids, natural or synthetic derivatives of vitamin A, are effective in the treatment of acute promyelocytic leukemia (APL) and are used in the chemoprophylaxis of cancers such as breast, skin, head and neck and liver cancers. Retinoid bound to the promoter of TRIM31 to induce TRIM31 expression and then suppressed the proliferation of breast cancer (31). MicroRNA is a small single-stranded noncoding RNA with a length of 21-23 nt that regulates the transcriptional inhibition, cleavage and degradation of mRNA (32). According to related studies, abnormal miRNA function is closely related to tumor invasion and metastasis (33, 34). In ovarian cancer, microRNA-551b downregulates TRIM31 expression by targeting its 3’ UTR to promote cancer progression (35). In addition, microRNA-29c-3p is abnormally expressed in many cancers, including gastric cancer, colon cancer, pancreatic cancer and hepatocellular carcinoma (36–38). Overexpression of microRNA-29c-3p significantly inhibited the proliferation and migration of hepatocellular carcinoma (HCC) cells *in vitro* and the growth of HCC tumors *in vivo*. Mechanistically, microRNA-29c-3p directly bound to the TRIM31 promoter and suppressed TRIM31 expression (39).

Posttranslational modifications including phosphorylation, ubiquitination and acetylation have been shown to modulate various biological functions, such as cell signal conduction, protein–protein interactions, protein transport, cell differentiation and proliferation through regulating the protein conformation, localization, stability and activity (40, 41). The TRIM31 protein is polyubiquitinated in gastric cancer, which leads to its proteasomal degradation. Furthermore, the ubiquitin proteasome-regulated degradation of TRIM31 was confirmed in AsPC-1 pancreatic cancer cells (23). These discoveries suggest that posttranslational modification can control the abundance of endogenous TRIM31.

## 4 The dual role of TRIM31 in cancer: Oncogene or Tumor Suppressor?

### 4.1 The tumor suppressor role of TRIM31

**Figure 3:**
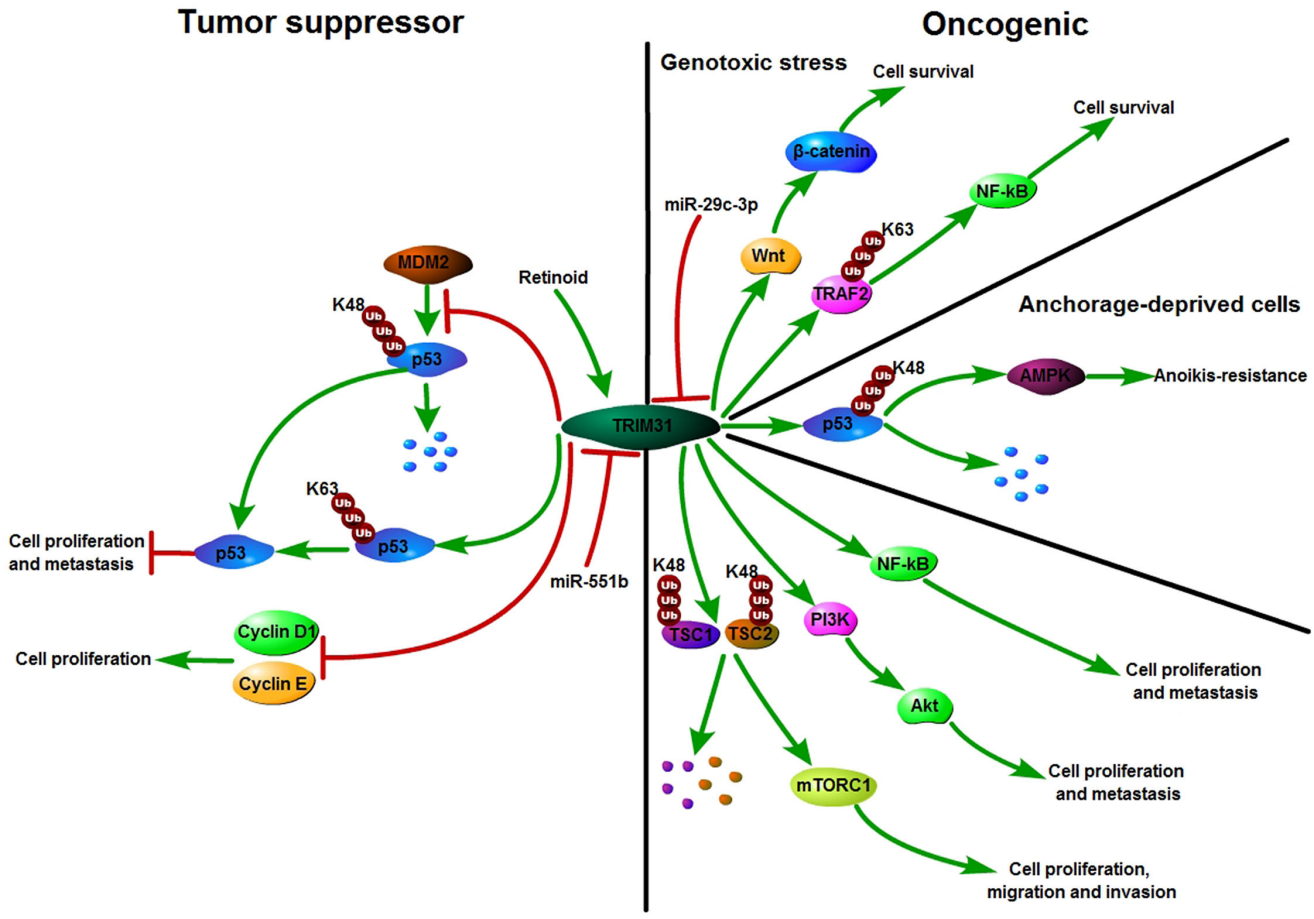
Schematic diagram of TRIM31 function in cancer. TRIM31 can act as either a tumor suppressor or an oncogene. TRIM31 inhibits cell growth by reducing the expression of Cyclin D1 and Cyclin E. TRIM31 stabilizes P53 expression to suppress tumor cell proliferation and metastasis. TRIM31 promotes tumor onset and progression by regulating the P53, mTORC1, PI3K-Akt, NF-kB and Wnt/β-catenin pathways. Retinoid induced cell growth arrest by targeting the promoter of TRIM31. miR-551b suppresses the expression of TRIM31 by targeting its 3’-UTR and further promotes cell invasion and drug resistance. miR-29c-3p can inhibit TRIM31 expression by targeting its 3’-UTR. TRIM31 activated Wnt/β-catenin signaling to promote cell survival.

Recently, increasing evidence has shown that TRIM31 plays an important tumor suppressor role in the occurrence and development of various cancers. TRIM31 was first reported in breast cancer in 2002. Retinoid induced proliferation inhibition of breast carcinoma cells by targeting the TRIM31 promoter (31). In our study, we found that TRIM31 directly interacted with p53 and subsequently stabilized and activated p53 by inducing K63-linked ubiquitination as well as inhibiting MDM2-mediated K48-linked ubiquitination of p53 and then suppressing breast cancer progression (22). In addition, TRIM31 plays a potential tumor suppressor role in non-small cell lung cancer. The expression of TRIM31 in lung cancer cell lines was lower than that in the normal bronchial cell line HBE. TRIM31 inhibited the cell proliferation rate and colony formation by reducing the expression of the cell cycle regulators cyclin D1 and cyclin E (30). Moreover, TRIM31 can be recognized as a growth suppressor at the early stage of gastric adenocarcinoma (23). Therefore, TRIM31 may act as a tumor suppressor in the early stage of the tumor. Altogether, these studies have shown that TRIM31 might play a tumor suppressor role in breast cancer, non-small cell lung cancer and the early stage of gastric adenocarcinoma (**Figure 3**).

### 4.2 The oncogene role of TRIM31

Recently, studies have shown that TRIM31 is an oncogene in various cancers. A number of carcinogenic mechanisms have been proposed for TRIM31, such as regulating the P53, mTORC1, PI3K-Akt, NF-kB and Wnt/β-catenin pathways to promote tumor onset and progression (24–30). P53 is one of the most important tumor suppressor proteins. In differentiated cells, the abundance and activity of p53 are strictly regulated. With an increased risk of acquiring mutations, the accumulation and activation of p53 protein are regulated by posttranslational modifications (42). TRIM31 was significantly upregulated in the anchorage-deprived HCC cells compared with their attached counterparts and promoted anoikis resistance. TRIM31 can directly interact with p53, which is an inhibitor of the AMPK pathway, and regulate the K48-linked ubiquitous degradation of p53. It was further confirmed that excessive activation of the AMPK pathway is the cause of TRIM31-mediated HCC cell resistance to anoikis. That is, TRIM31 facilitates anoikis resistance by targeting the degradation of p53 and subsequently overactivating the AMPK pathway (24). AMPK phosphorylates tuberous sclerosis complex 2 (TSC2) and enhances its activity (43). TSC2 is an upstream inhibitor of the mTORC1 pathway. MTOR forms two unique catalytic subunits of the complex, called mTORC1 and mTORC2, and it plays critical roles in a variety of biological processes, such as cell growth, survival, autophagy, metabolism, and immunity (44, 45). TRIM31 facilitates the malignant behaviors of HCC cells by overactivating the target of the mTORC1 pathway. Further studies have shown that TRIM31 plays a carcinogenic role by directly interacting with the TSC1 and TSC2 complexes and facilitating the ubiquitination of K48 and the degradation of the complex (19).

The phosphoinositide 3-kinase (PI3K)–AKT pathway is an important node in controlling cell growth, migration, proliferation, and metabolism in mammalian cells and is the most commonly activated pathway in human cancers (46). In glioma, by silencing or overexpressing TRIM31 expression, the proliferation, invasion and migration of glioma cells could be downregulated or upregulated through the PI3K/Akt signaling pathway (27). Moreover, TRIM31 can activate the PI3K/Akt signaling pathway to enhance chemoresistance in glioblastoma (47). In gallbladder cancer, TRIM31 promotes proliferation and invasion *via* the PI3K/Akt signaling pathway (25). The PI3K/AKT/IKK alpha pathway regulates the activation of NF kappa B and β-catenin in CRC cell lines (48). Nuclear factor kappa B (NF-κB) is activated in various cancers and not only coordinates with immunity and inflammation but also plays a vital role in the development of cancer (49, 50).. Past research has shown that ubiquitin modification plays an important role in the regulation of NF-κB signaling (51–53). As an E3 ubiquitin ligase, TRIM31 promotes K63-linked polyubiquitination of tumor necrosis factor receptor-associated factor 2 (TRAF2) to upregulate the levels of nuclear p65 and then maintains the activation of NF-κB in pancreatic cancer cells. Furthermore, TRIM31 promotes gemcitabine resistance in pancreatic cancer cells by activating the NF-κB signaling pathway (28). In addition, TRIM31 activates the NF-κB pathway to promote migration and invasion in glioma and colorectal cancer (26, 54). TRIM31 regulates Wnt/β-catenin signaling to promote acute myeloid leukemia progression and sensitivity to daunorubicin (29). Collectively, TRIM31 plays an oncogene role in cancer by regulating the p53, mTORC1, NF-κB, and PI3K-Akt pathways (**Figure 3**).

### 4.3 The possible mechanism of TRIM31 in promoting or suppressing cancer

TRIM31 is a critical factor that is able to perform multiple functions in cancer, and the complex function of TRIM31 makes it difficult to identify TRIM31 as an oncogene or tumor suppressor. Here, we will discuss why TRIM31 can promote or inhibit cancer from the following aspects. First, TRIM genes are usually expressed in a variety of splicing forms (55). There are three isoforms of TRIM31 (TRIM31α, TRIM31β and TRIM31γ). TRIM31α was the most common splicing form and has been registered in the public database. TRIM31 β is a truncated form of TRIM31 at the C-terminus. TRIM31 γ is a mutant protein of TRIM31 truncated at the C-terminus. Studies have shown that the TRIM31 isoforms have different biological roles in cancer. Therefore, the differential expression of TRIM31 isoforms may lead to the different roles of TRIM31 in cancer. Second, proteins containing a RING finger domain can serve as E3 ubiquitin ligases (17), and Sugiura proved that TRIM31 has autoubiquitylating activity *in vitro*. It has been demonstrated that the autoubiquitination activity of TRIM31 regulates the intracellular abundance of TRIM31 (23). The strict regulation of the TRIM31 protein level may be linked to its seemingly contradictory behaviors in the cancer process. Third, several cancer-associated proteins that can be posttranslationally regulated by TRIM31 have been reported, such as TRAF2, TSC1/TSC2 and P53. The different target proteins of TRIM31 may decide the tumor promoter or tumor suppressor of TRIM31 in cancer. Therefore, discovering new target proteins of TRIM31 is necessary to further understand the role of TRIM31 in cancer.

## 5 TRIM31: Growing influence in innate immunity and autophagy

### 5.1 The emerging role of TRIM31 in innate immunity

Innate immunity provides the first line of defense against invading pathogens. Activation of innate immunity requires the recognition of pathogen-associated molecular patterns through pattern-recognition receptors (56, 57). As a regulator of Mitochondrial antiviral signaling protein (MAVS) aggregation, TRIM31 can be recruited to mitochondria after viral infection and specifically regulate antiviral signaling mediated by RIG-I-like receptor (RLR) pattern-recognition receptors. Further study showed that TRIM31 interacted with MAVS and catalyzed the Lys63 (K63)-linked polyubiquitination of MAVS by Lys10, Lys311 and Lys461. This modification promoted the formation of prion-like aggregates of MAVS after viral infection (20). Moreover, USP18 interacts with TRIM31 to promote the K63-linked polyubiquitination of MAVS and then positively regulates innate antiviral immunity (58). PB1-F2, Rac1, PRMT7 and FAF1 disrupt TRIM31 interaction with MAVS to inhibit MAVS activation and negatively regulate innate antiviral immune responses (59–62). HBV (hepatitis B virus) infection was reported to induce type III IFNs, and TRIM31 was found to be a type III IFN-stimulated gene. IFN-induced TRIM5γ recruits TRIM31 to degrade HBx, resulting in suppression of hepatitis B virus replication (63, 64). In addition to its crucial role in antiviral processes, TRIM31 also has an important role in promoting viral infection. COVID-19 caused by the novel severe acute respiratory syndrome (SARS) coronavirus 2 (SARS-CoV-2) is rapidly emerging and spreading worldwide. Wang et al. reported that the dimeric domain protein (SARS2-NP) of the SARS-CoV-2 nucleocapsid is required for liquid-liquid phase separation of SARS2-NP and RNA, which suppresses Lys63-linked polyubiquitination and aggregation of MAVS by reducing TRIM31 binding to MAVS, thus inhibiting the innate antiviral immune response (65). Moreover, Temena et al. also found that TRIM31 is positively correlated with SARS−CoV−2 associated genes TMPRSS2−TMPRSS4 and knockdown of TRIM31 significantly altered viral replication and viral processes in gastrointestinal cancer samples. This result suggests that TRIM31 may play a role in increasing the susceptibility to SARS-CoV-2 viral infection in patients with gastrointestinal cancers (66).

The NLRP3 inflammasome is a multiprotein platform that comprises NLRP3, ASC, and caspase-1 and plays crucial roles in host defense against pathogens. The NLRP3 inflammasome is involved in many kinds of diseases, such as cancer, gout, autoimmune disorders, atherosclerosis, type 2 diabetes and obesity (67–70). TRIM31 has been reported to be a feedback suppressor of the NLRP3 inflammasome. TRIM31 directly binds to NLRP3 and promotes K48-linked polyubiquitination and proteasomal degradation of NLRP3. Furthermore, TRIM31 deficiency attenuates the severity of dextran sodium sulfate (DSS)-induced colitis, an inflammatory bowel disease model in which NLRP3 exerts a protective effect (21). Moreover, AKT bound to NLRP3 and phosphorylated it on S5. This phosphorylation event also stabilized NLRP3 by reducing its ubiquitination on lysine 496, which inhibits its proteasome-mediated degradation by TRIM31 (71). In addition, TRIM31 promoted the ubiquitination of NLRP3 to alleviate IL−1ß secretion and diminished the development of apical periodontitis (72). TRIM31 also inhibited the NLRP3 inflammasome and pyroptosis through ubiquitination of NLRP3 in retinal pigment epithelial cells (73). CRNDE interacted with NLRP3 and decreased TRIM31-mediated NLRP3 ubiquitination to activate the NLRP3 inflammasome and exacerbate IgA nephropathy progression (74). In addition to the NLRP3 inflammasome, TRIM31 also plays a crucial role in fungal infections. TRIM31 regulates antifungal immunity by facilitating K27-linked polyubiquitination of SYK (75).

### 5.2 TRIM31 and autophagy

**Figure 4:**
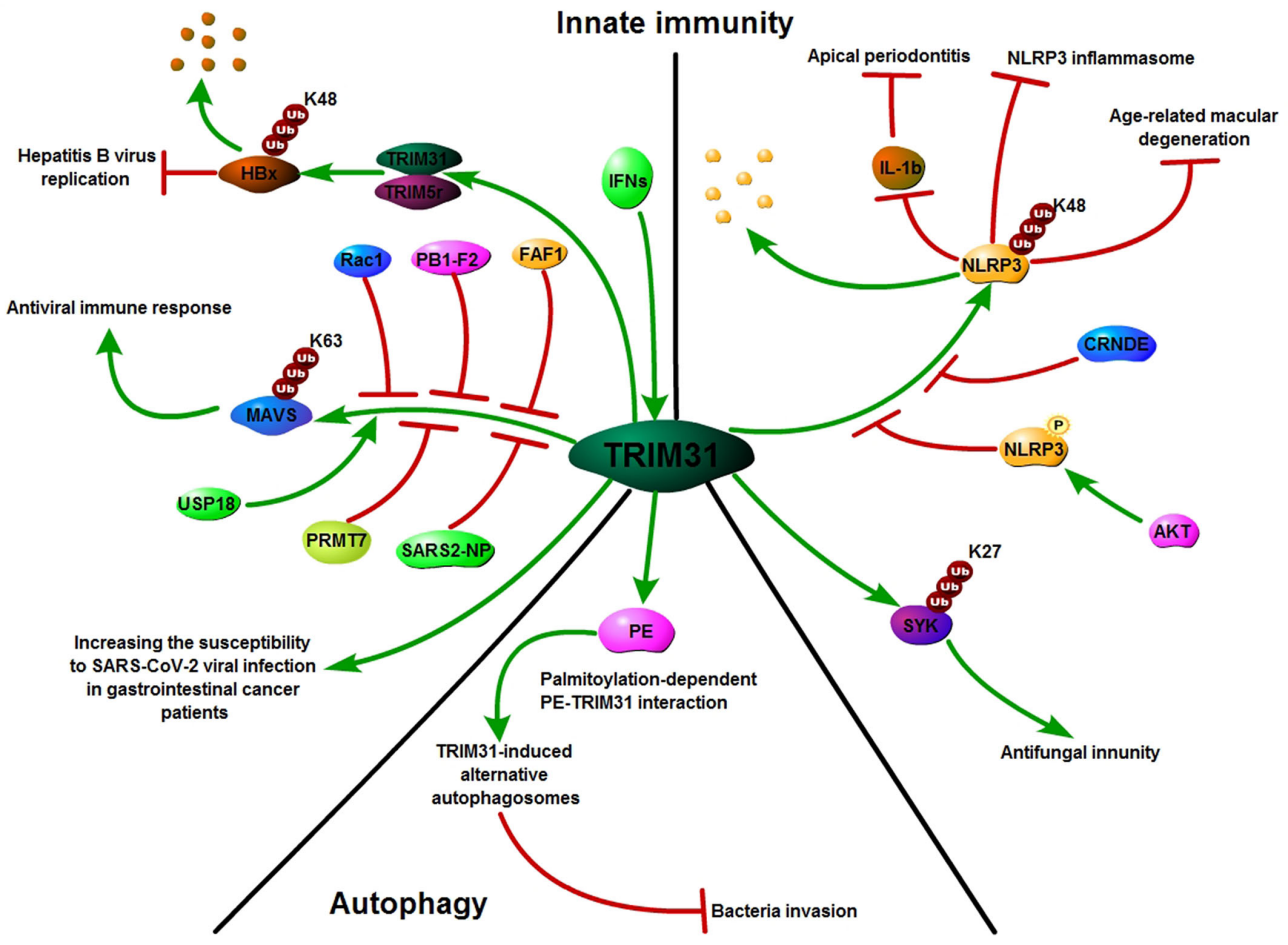
TRIM31: Growing influence in innate immunity and autophagy. TRIM31 plays an important role in innate immunity; TRIM31 interacts with MAVS and catalyzes the Lys63 (K63)-linked polyubiquitination of MAVS to promote the formation of prion-like MAVS aggregates after viral infection. USP18 promotes TRIM31-mediated K63-linked MAVS polyubiquitination, while PB1-F2, Rac1, PRMT7, SARS2-NP and FAF1 inhibit TRIM31-mediated K63-linked MAVS polyubiquitination. TRIM31 directly binds to NLRP3 and promotes K48-linked polyubiquitination and proteasomal degradation of NLRP3. AKT and CRNDE decreased TRIM31-mediated NLRP3 ubiquitination. Alongside its emerging role in innate immunity, TRIM31 is also known to be involved in autophagy.

Autophagy is one of the major intracellular degradation systems in addition to the ubiquitin–proteasome system. A primary role of autophagy is to maintain cellular homoeostasis by degrading intracytoplasmic proteins and organelles through starvation and by recycling multiple sources (76–78). Recent studies have shown that several TRIM proteins regulate cancer progression *via* autophagy. TRIM59 inhibits p62 selective autophagy degradation of PDCD10 to promote the motility of breast cancer (79). In A549/DDP cells, knockdown of TRIM65 can inhibit autophagy and cisplatin resistance by regulating miR-138-5P/ATG7 (80). TRIM31, an intestine-specific protein localized in mitochondria, is essential for promoting lipopolysaccharide-induced Atg5/Atg7-independent autophagy. TRIM31 directly interacts with phosphatidylethanolamine in a palmitoylation-dependent manner, leading to the induction of autolysosome formation (81). Altogether, TRIM31 plays an important role in innate immunity, autophagy and other human pathologies (**Figure 4**).

## 6 Conclusion and future perspectives

In conclusion, although the function of TRIM31 has been studied for many years, there is still much to be clarified regarding the role of TRIM31 in cancer. A number of studies suggest that TRIM31 may serve as an oncogene when it is highly expressed and may facilitate cancer progression, metastasis and drug resistance by inducing the mTORC1 pathway (19), activating the NF-κB and AKT signaling pathways (25–28), and downregulating the activity of p53 (24). However, some studies have shown that TRIM31 can act as a tumor suppressor, and its high expression inhibits the proliferation and metastasis of cancer by stabilizing p53 or decreasing the expression of cyclin D1 and cyclin E. Although TRIM31 has been extensively studied, some questions should also be considered. First, the regulation of TRIM31 in cancer should be further researched. Recent research has shown only that TRIM31 can be regulated by posttranslational modification, and whether there are other regulatory mechanisms is still unclear. Second, the mechanism by which TRIM31 promotes and suppresses cancer needs further study. For example, whether TRIM31 regulates cancer progression *via* autophagy or innate immunity is unknown. Third, as an E3 ubiquitin ligase, TRIM31 can target many proteins (**Table 1**). Therefore, it is very important to find new target proteins for studying the function of TRIM31 in cancer. Although there are still many questions to be addressed, we believe that in-depth understanding of the TRIM31 in carcinogenesis may help to answer whether TRIM31 possesses the potential to become a new anticancer target.

**Table 1:** The TRIM31 targets or interacting proteins.

Target or interacting proteins	Modification	Effect	Outcome	Reference
P53	K48-linked poly-ubiquitination	Degradation of p53	Promoting the anoikis-resistance	(24)
TSC1/TSC2	K48-linked poly-ubiquitination	Degradation of TSC1/TSC2	Promoting HCC progression	(19)
TRAF2	K63-linked poly-ubiquitination	Activation of NF-κB	Promoting the gemcitabine resistance	(28)
P53	K63-linked poly-ubiquitination	Activation of p53	Suppressing the proliferation and migration of breast cancer cell	(22)
MAVS	K63-linked poly-ubiquitination	Promoting the formation of prion-like aggregates	Activation of antiviral immunity	(20)
USP18	No	Promoting the K63-linked polyubiquitination of MAVS	Activation of antiviral immunity	(58)
HBx	K48-linked poly-ubiquitination	Degradation of HBx	Inhibiting HBV Replication	(62)
NLRP3	K48-linked poly-ubiquitination	Degradation of NLRP3	Attenuating NLRP3 inflammasome activation	(70)
SYK	K27-linked poly-ubiquitination	Activation of SYK	Promoting antifungal immunity	(74)

Currently, pharmaceutical companies have entered the era of E3 ubiquitin ligase-targeted therapy, and targeting the E3 ligase is gradually becoming a considerable cancer treatment option (82). Proteolysis-targeting chimera (PROTAC) has been developed as a useful protein-targeted degradation technique. A bifunctional PROTAC molecule is composed of a ligand of the protein of interest (POI) and a covalently linked ligand of an E3 ubiquitin ligase (E3). Upon binding to POI, PROTAC can recruit E3 for POI ubiquitination, which is subject to proteasome-mediated degradation (83). However, the PROTAC technique has not been applied to the TRIM31 protein, and future studies may focus on the application of PROTAC to TRIM31 protein.

## Author contributions

Conception and design: YG and PL. Initial manuscript writing: YG. Confirmation of Manuscript: PL, YH and CW. All authors contributed to the article and approved the submitted version.
